# A simple and cost-effective transformation system for *Porphyromonas gingivalis* via natural competence

**DOI:** 10.3389/fmicb.2024.1476171

**Published:** 2024-10-21

**Authors:** Kimihiro Abe, Hiroko Yahara, Ryoma Nakao, Takehiro Yamaguchi, Yukihiro Akeda

**Affiliations:** ^1^Department of Bacteriology I, National Institute of Infectious Diseases, Tokyo, Japan; ^2^Research Center for Drug and Vaccine Development, National Institute of Infectious Diseases, Tokyo, Japan; ^3^Genome Medical Science Project, Research Institute, National Center for Global Health and Medicine, Tokyo, Japan

**Keywords:** *Porphyromonas gingivalis*, natural competence, transformation, horizontal gene transfer, genetic engineering

## Abstract

*Porphyromonas gingivalis* is a major oral bacterial pathogen responsible for severe periodontal diseases. Numerous studies have used genetic approaches to elucidate the molecular mechanisms underlying its pathogenicity. Typically, electroporation and conjugation are utilized for mutagenesis of *P. gingivalis*; however, these techniques require specialized equipment such as high-voltage electroporators, conjugative plasmids and donor strains. In this study, we present a simple, cost-effective transformation method for *P. gingivalis* without any special equipment by exploiting its natural DNA competence. *P. gingivalis* ATCC 33277 was grown to the early-exponential phase and mixed with a donor DNA cassette. This mixture was then spotted onto a BHI-HM blood-agar plate and incubated for one day to promote colony biofilm formation. The resulting colony biofilm was suspended in a liquid medium and spread onto antibiotic-containing agar plates. Transformants appeared within 4 to 5 days, achieving a maximum efficiency of 7.7 × 10^6^ CFU/μg. Although we optimized the transformation conditions using a representative strain ATCC 33277, but the method was also effective for other *P. gingivalis* strains, W83 and TDC60. Additionally, we discovered that deletion of *PGN_0421* or *PGN_0519*, encoding putative ComEA and ComEC, abolished competency, indicating that these gene products are essential for the natural competence.

## 1 Introduction

Genetic transformation is a key technique in modern DNA recombination technology, used to understand gene functions by altering an organism’s phenotypes or creating genetically modified organisms of industrial or clinical importance. Effective transformation systems and genetic tools enhance research and broaden the applications of genetic engineering. *Escherichia coli* and *Bacillus subtilis*, representing Gram-negative and Gram-positive bacteria respectively, are the most extensively studied bacterial species in genetics, biochemistry, and genetic engineering. This prominence stems partly due to the invention of divalent metal cation-induced competent cells for *E. coli* (Mandel and Higa, 1970) and the discovery of natural competence in *B. subtilis* (Spizizen, 1958) in the early days of bacteriology. Research into genetic transformation remains important and challenging, especially for many non-model organisms.

*Porphyromonas gingivalis* is a non-motile Gram-negative anaerobic bacterium that inhabits the oral cavity and is a keystone pathogen responsible for serious periodontitis among many oral bacteria (Sharaf and Hijazi, 2022; Morrison et al., 2023). Recent evidence suggests that *P. gingivalis* may also be linked to various systemic diseases commonly found in older adults, including diabetes, Alzheimer’s disease, and cardiovascular diseases (Jungbauer et al., 2022; Lamont et al., 2022; Li et al., 2022; Wang et al., 2022). As developed countries confront the challenge of an aging population, there is an urgent need for a better understanding of pathogenicity of *P. gingivalis*. Genomic analyses have revealed that *P. gingivalis* possesses various (putative) virulence factors, including proteases, collagenase, hemolysins, endotoxins, and adhesins (Nelson et al., 2003; Naito et al., 2008; Watanabe et al., 2011). However, the complete picture of *P. gingivalis* pathogenicity remains unclear. Genetic approaches, such as reverse genetics and genome editing, provide powerful tools for gaining a deeper understanding of the molecular mechanisms underlying the pathogenicity.

Introducing exogenous DNA into bacteria is typically achieved through chemically induced competent cells (chemical competent cells), bacteriophage transduction, conjugation, electroporation, or natural competence (Liu et al., 2022). To date, however, there are no reports of using chemical competent cells or phage transduction for *P. gingivalis*. Currently, conjugation and electroporation are employed for the genetic transformation of *P. gingivalis* (Dyer et al., 1992; Yoshimoto et al., 1993; Smith, 1995; Bélanger et al., 2007). Conjugation effectively delivers DNA into *P. gingivalis*, but it relies on conjugative plasmids like pT-COW (Gardner et al., 1996) and specialized donor strains like *E. coli* S17-1 (Simon et al., 1983). Electroporation can introduce both linearized DNA and circular plasmids into bacterial cells but requires a high-voltage electroporator.

Natural competence refers to the ability of bacteria to actively incorporate exogenous DNA into the cells. Typically, naturally competent bacteria employ a species-specific DNA-uptake machinery for DNA transport (Seitz and Blokesch, 2013). Genetic transformation via natural competence is particularly appealing for genetic manipulation because it does not require any special equipment or materials. The natural competence of *P. gingivalis* was first reported by Tribble et al. (2012), who studied conjugative transfer of chromosomal DNA in *P. gingivalis* biofilms (Tribble et al., 2007). They discovered that *P. gingivalis* acquires DNA competence in biofilms and that a cytoplasmic protein, ComF, is necessary for this process, although the exact mechanism remains unclear.

In this study, we report a straightforward and cost-effective transformation method for *P. gingivalis* that leverages its natural competence. We detail the optimized procedure and experimental conditions, making this technique accessible for routine use in any laboratory focused on *P. gingivalis* research.

## 2 Materials and methods

### 2.1 Bacterial strains and culture conditions

Bacterial strains are listed in Supplementary Table S1. *P. gingivalis* strains were cultured at 37°C in 5 mL of Brain-Heart Infusion medium (Becton, Dickinson and Company, Maryland, United States) supplemented with 5 μg/mL hemin and 1 μg/mL menadione (referred to as BHI-HM) under anaerobic conditions: 10% H_2_, 10% CO_2_, and 80% N_2_ (Nakao et al., 2006). BHI-HM blood-agar plates were prepared by adding 5% defibrinated sheep blood and 1.5% (w/v) agar into BHI-HM. To select transformants, the medium was supplemented with 10 μg/mL erythromycin or 1 μg/mL ampicillin.

### 2.2 Preparation of donor DNA

Primers used in this study are listed in Supplementary Table S2. An erythromycin resistant gene, *ermF*, was generated by PCR using the PKB-301/302 primer set and pHS17 (Haake et al., 2000) as the template DNA. DNA fragments of 0.5 kb corresponding to the upstream and downstream regions of *PGN_0032*–*PGN_0033* were amplified with primer sets PKB-440/418 (500-bp upstream homology arm) and PKB-419/441 (500-bp downstream), respectively. These fragments were assembled into the *ermF* cassette by overlap extension PCR with primers PKB-440/441. The upstream and downstream homology arms ranging from 50 to 2,000 bp were generated using the following primer sets: PKB-446/418 (50-bp arm), PKB-444/418 (100-bp arm), PKB-442/418 (250-bp arm), PKB-428/418 (1,000-bp arm), and PKB-417/418 (2,000-bp arm) for the upstream homology arms; PKB-419/447 (50-bp arm), PKB-419/445 (100-bp arm), PKB-419/443 (250-bp arm), PKB-419/429 (1,000-bp arm), and PKB-419/420 (2,000-bp arm) for the downstream homology arms.

### 2.3 Transformation assay

**Figure 1:**
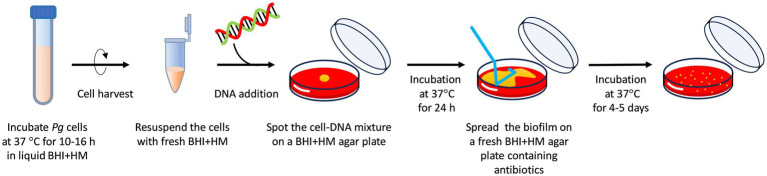
Schematic of the transformation procedure. (i) Culture *P. gingivalis* at 37°C in 5 mL of BHI-HM under anaerobic conditions until the cell growth reaches the early-mid exponential phase (at OD_600_ of approximately 0.3; 10–16 h of cultivation). (ii) Harvest the cells by brief centrifugation and resuspend the pellet in 500 μL of fresh, prewarmed BHI-HM. (iii) Mix 20 μL of the cell resuspension with 100 ng of donor DNA containing 1,000-bp homology arms, spot the mixture on a BHI-HM blood-agar plate, and incubate at 37°C for 24 h under anaerobic conditions. (iv) Collect and suspend the colony biofilm in 500 μL of fresh, prewarmed BHI-HM and spread the suspension on an antibiotic-containing BHI-HM blood-agar plate. (v) Incubate the plate at 37°C under anaerobic conditions for 4–5 days. These are the optimized conditions for transformation determined in this study.

Schematic of the transformation assay is shown in Figure 1. The optimized procedure and conditions are outlined here, with different conditions indicated in the results section or figure legends. *P. gingivalis* strains were anaerobically cultured at 37°C in 5 mL of BHI-HM until reaching the early-mid exponential phase. The cells were harvested by brief centrifugation at 8,000 g for 4 min at room temperature. After removing the supernatant, the cell pellet was suspended in 500 μL of fresh prewarmed BHI-HM. Twenty microliters of the cell suspension were mixed with 100 ng of donor DNA and spotted onto a BHI-HM blood-agar plate lacking erythromycin or ampicillin. The plate was incubated anaerobically at 37°C for 24 h, after which a colony biofilm was scraped with a disposable inoculating 10-mm loop from the plate and suspended in 500 μL of fresh pre-warmed BHI-HM by the stirring the loop (the biofilm is non-sticky and can be easily removed from the loop and suspended in the liquid medium). The colony suspension was serially diluted in BHI-HM and spread onto BHI-HM blood-agar plates containing the appropriate antibiotics. Then, the plates were incubated at 37°C for 5 days under anaerobic conditions. Transformation efficiency was assessed by counting the number of the antibiotic-resistant colonies.

### 2.4 Construction of *Porphyromonas gingivalis* mutant strains

To create the deletion mutant for *comEA* (*PGN_0421*), DNA fragments corresponding to the 5′- and 3′-flanking regions of the gene were amplified from the genome of *P. gingivalis* ATCC 33277 by PCR using the primer sets PKB-462/455 and PKB-456/463, respectively. The ampicillin resistant gene, *cepA*, was amplified from *P. gingivalis* KDP501 (Sato et al., 2018) using the primer set PKB-421/422. The DNA fragments were assembled to a Δ*comEA*::*cepA* cassette by overlap extension PCR with primers PKB-462/463. The resulting PCR product (100 ng) was introduced into *P. gingivalis* ATCC33277 via natural competence, as described above. Transformants were selected using 1 μg/mL ampicillin.

For the *comEC* (PGN_0519) deletion mutant, the 5′- and 3′-flanking regions were amplified using the primer sets PKB-460/451 and PKB-452/461, respectively. Preparation of the *cepA* cassette and transformation were carried out following the same procedure as used for the *comEA* mutant.

## 3 Results

### 3.1 Design of experimental procedure for *Porphyromonas gingivalis* transformation

A previous study reported the natural competence of *P. gingivalis* mentioning that biofilm conditions appear to enhance extracellular DNA uptake (Tribble et al., 2012). Based on the study, we designed a transformation protocol for *P. gingivalis*, as shown in Figure 1. Recipient cells were propagated by cultivating them in a liquid BHI-HM medium. Donor DNA was added to the recipient cells and then spotted onto a BHI-HM blood-agar plate without antibiotics to facilitate colony biofilm formation and DNA uptake. The colony biofilm was suspended in liquid BHI-HM and spread onto an antibiotic-containing BHI-HM blood-agar plate to select transformants.

### 3.2 Transformation assay

**Figure 2:**
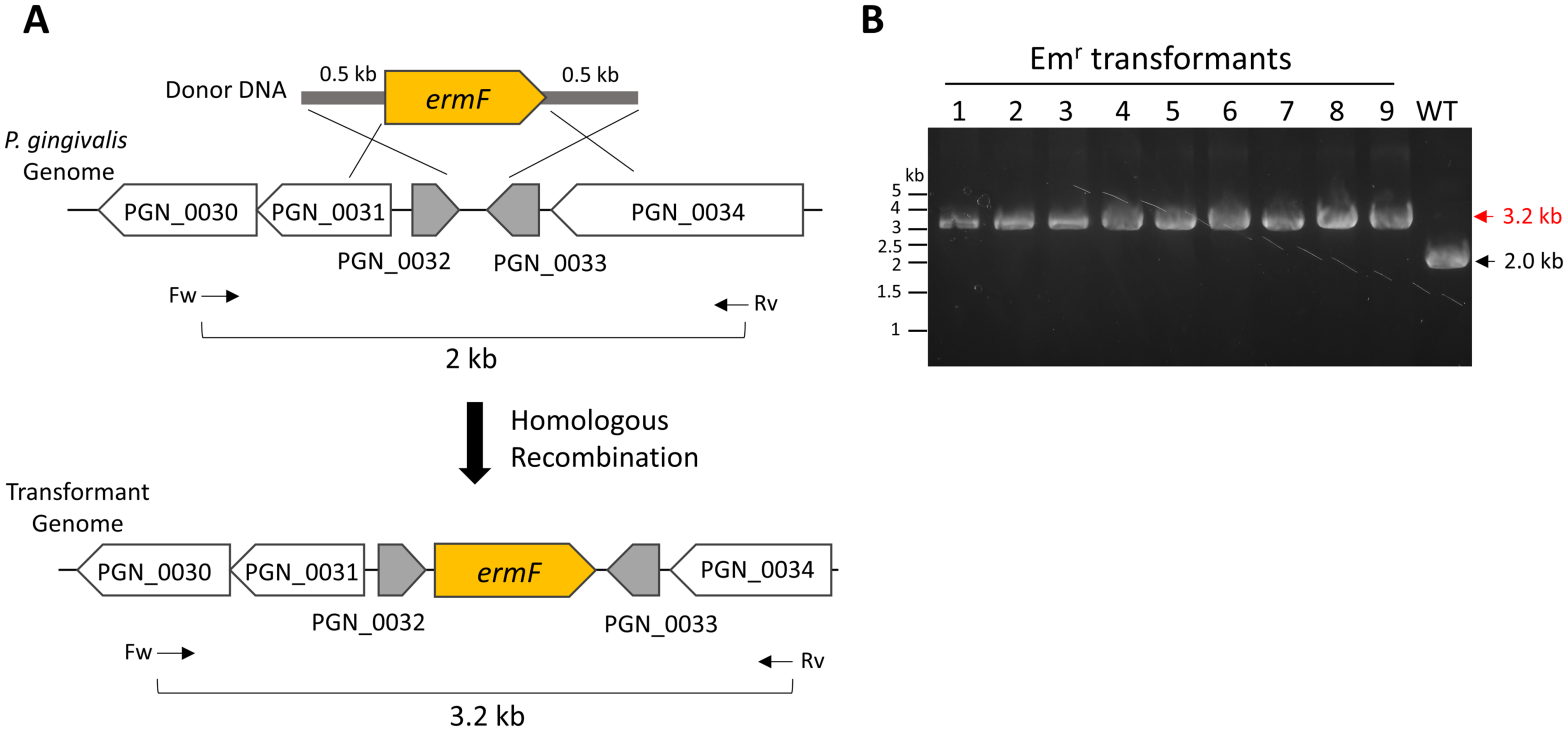
Natural competence-mediated integration of *ermF* into *P. gingivalis* genome. **(A)** Schematic representation of the integration of an erythromycin-resistant gene (*ermF*)-harboring donor DNA into the *P. gingivalis* ATCC 33277 genome via double homologous recombination. The *ermF* donor DNA includes 500-bp homology arms for integration into the intergenic region between *PGN_0032* and *PGN_0033*. Arrows indicate the positions and orientations of PCR primers used for genotyping. **(B)** Colony PCR confirmation. The integration of *ermF* in the transformant genome was confirmed by colony PCR, examining nine colonies with the primers shown in **(A)**. A wild-type colony served as a negative control.

First, we prepared donor DNA containing an erythromycin-or ampicillin-resistant genes (*ermF* or *cepA*) for transformation. The intergenic region between *PGN_0032* and *PGN_0033* in the *P. gingivalis* ATCC 33277 genome was designated as the donor integration site to minimize the impact on cell viability and growth. The donor DNA fragments were flanked by 500-bp homology arms at their 5′ and 3′ ends, corresponding to the upstream and downstream regions of the integration site (Figure 2A).

To test our transformation method, *P. gingivalis* ATCC 33277 recipient cells were cultured at 37°C for 1 day in liquid BHI-HM. The recipient cells were mixed with 0.5 μg of *ermF* donor DNA, and a colony biofilm was developed for 24 h on a BHI-HM blood-agar plate. Afterward, the colony biofilm suspension was spread onto an erythromycin-containing BHI-HM blood-agar plate. Tiny colonies appeared on the plate 3 days after incubation. Five days after incubation, when the colonies had grown larger, colony PCR was performed to verify donor integration accuracy. In this PCR genotyping, a 3.2-kb band signal was detected when *ermF* was inserted into the intergenic region between *PGN_0032* and *PGN_0033*, while a 2.0-kb band was detected in the wild-type strain. All nine transformant colonies examined confirmed that *ermF* was correctly inserted into the intergenic region between *PGN_0032* and *PGN_0033* (Figure 2B, Em^r^ transformants No. 1–9). The integration had no significant effect on the cell growth, as expected (Supplementary Figure S1). Additionally, instead of *ermF*, *cepA* donor DNA was also successfully integrated into the intergenic region (Supplementary Figure S2). We attempted transformation by electroporation following the method described by Bélanger et al. (2007). However, we could not obtain any electroporation-mediated transformants, whereas the natural competence-mediated transformation produced >1 × 10^3^ Em^r^ transformants under the same conditions for donor DNA amount and cultivation. These results demonstrate the efficacy of our transformation method.

### 3.3 Influences of incubation time on DNA competency

**Figure 3:**
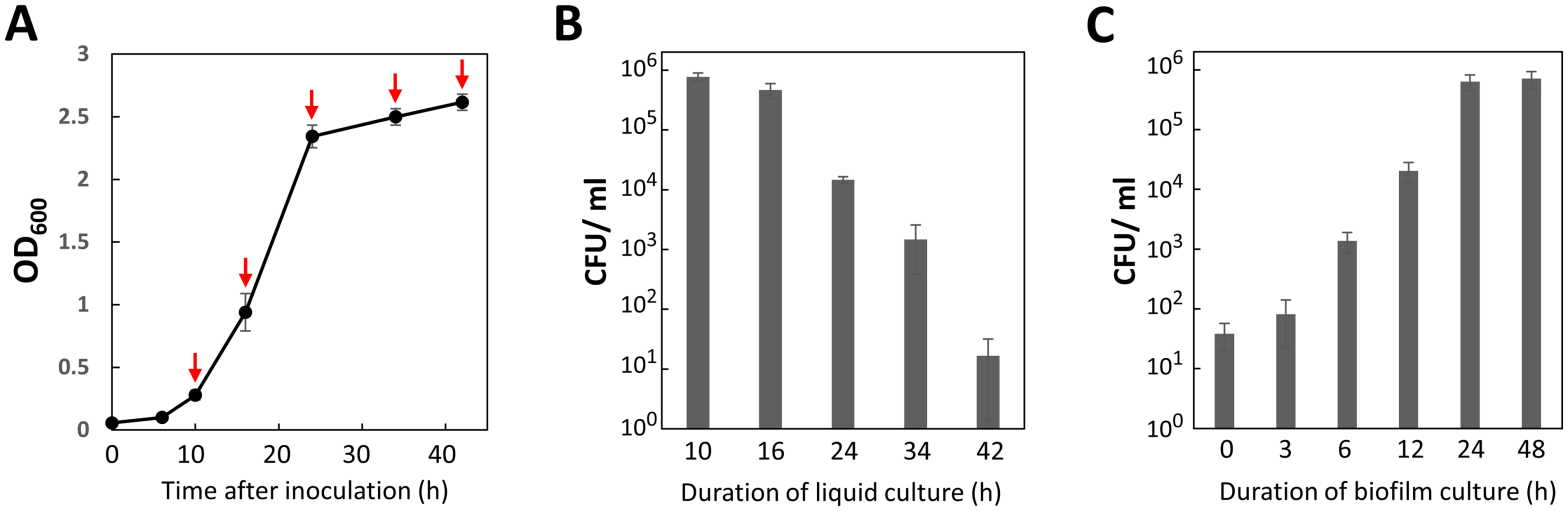
Effects of culture duration on transformation efficiency. **(A)** Cell growth. An optical density at 600 nm of *P. gingivalis* ATCC 33277 cultured at 37°C in liquid BHI-HM was measured at 6, 10, 16, 24, 34, and 42 h after inoculation. **(B)** Impact of cell growth phase on transformation efficiency. Cells harvested at the time points indicated by red arrows in **(A)** were transformed with 100 ng of *ermF* donor DNA with 1,000-bp homology arms. The colony biofilm was developed for 24 h. Transformation efficiency was assessed as the number of erythromycin-resistant (Em^r^) colonies per ml of the biofilm suspension (CFU/ml). Error bars represent ±standard deviations from three independent experiments. **(C)** Effect of biofilm culture duration on transformation efficiency. *P. gingivalis* recipient cells in the early-mid exponential phase were mixed with 100 ng of donor DNA with 1,000-bp homology arms and spotted on agar plates. Colony biofilms collected at the specified time points were spread on BHI-HM blood-agar plates containing erythromycin. Transformation efficiency was quantified as CFU/ml of Em^r^ colonies. Error bars represent ±standard deviations from three independent experiments.

In general, the expression of natural competence is tightly regulated and temporal (Salvadori et al., 2019). Therefore, we investigated the relationship between transformation efficiency and the growth phase of the recipient cells. Under our culture conditions, the lag phase lasted 6 h after inoculation. The cell culture then entered the exponential phase by 10 h and reached the stationary phase at 24 h (Figure 3A). *P. gingivalis* recipient cells were harvested at 10, 16, 24, 34, and 42 h post-inoculation (Figure 3A, red arrows). For this transformation assay, we used 100 ng of *ermF* donor DNA with 1,000-bp homology arms. As shown in Figure 3B, the highest transformation efficiency was 7.7 × 10^5^ CFU/mL of the biofilm suspension (7.7 × 10^6^ CFU/μg of DNA) during the early-exponential phase. Given that the total viable cell count of the biofilm suspension was 3.9 × 10^9^ CFU/mL, the transformation frequency (the ratio of erythromycin-resistant colonies to total colonies) was 2.0 × 10^−4^. In contrast, transformation efficiency drastically declined upon entering the stationary phase (24 h and later time points). These results indicate that the natural competence of *P. gingivalis* ATCC 33277 is activated during the early growth phase in BHI-HM liquid culture.

Biofilm development is a crucial step for DNA uptake and the expression of ErmF, which confers antibiotic resistance. We investigated the effects of incubation duration of the *P. gingivalis* colony biofilm on the DNA competency (Figure 3C). Although a small number of transformants were generated by 0 to 3 h incubation for the biofilm development (3.8–8.2 × 10^1^ CFU/mL), it seemed that at least 6 h of incubation was necessary for stable performance in routine transformation experiments (Figure 3C, 6 h, 1.3 × 10^3^ CFU/mL). Transformation efficiency plateaued at 24 h incubation, with only a slight increase observed at 48 h. Considering the balance between transformation efficiency and the time required for the experiment, we concluded a 24-h culture as optimal for biofilm development.

### 3.4 Dependence of transformation efficiency on dose and arm length of donor DNA

**Figure 4:**
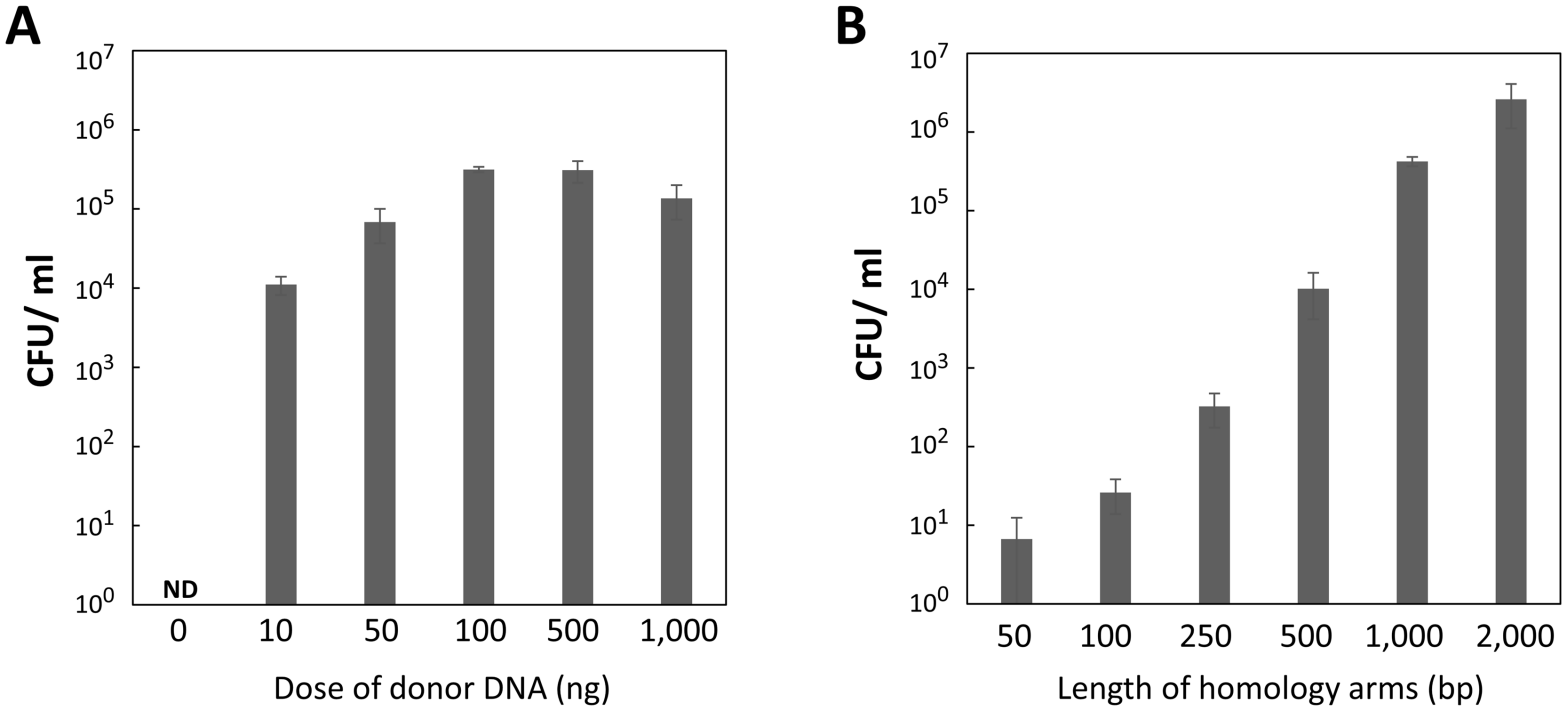
Influence of donor DNA amounts and homology arm length on transformation efficiency. **(A)** Donor DNA amounts. *P. gingivalis* recipient cells in the early-mid exponential phase were transformed with varying amounts of donor DNA containing 1,000-bp homology arms. Colony biofilms were developed for 24 h. Transformation efficiency was measured as CFU/ml of Em^r^ colonies. Error bars represent ±standard deviations from three independent experiments. ND, Not Detected. **(B)** Homology arm length. *P. gingivalis* recipient cells in the early-mid exponential phase were transformed with 100 ng of donor DNA containing various homology arm lengths. The colony biofilm was developed for 24 h. Transformation efficiency was quantified as CFU/ml of Em^r^ colonies. Error bars represent ±standard deviations from three independent experiments.

We investigated how transformation efficiency depends on the dose and length of homology arms in the donor DNA. Recipient cells in the exponential phase were mixed with 0, 10, 50, 100, 500, and 1,000 ng of donor DNA, each containing 1,000-bp homology arms. The transformation assay revealed a dose-dependent increase in transformation efficiency, peaking with 100 or 500 ng of donor DNA (Figure 4A). Even as little as 10 ng of donor DNA was sufficient to achieve 10^4^ CFU/mL, highlighting the high efficiency of our transformation method. However, adding 1,000 ng of donor DNA resulted in a slight decrease in efficiency, likely due to the dilution of recipient cells caused by the large volume of the DNA solution when spotted onto the agar plate.

Subsequently, we determined the minimal length of homology arms required for effective homologous recombination with the genomic DNA. Donor DNA with 50-, 100-, 250-, 500-, 1,000-, and 2,000-bp homology arms was tested. Transformation efficiency increased proportionally with the length of the homology arms (Figure 4B), showing more than a 10-fold increase as the length doubled. Unexpectedly, even 50-bp homology arms still produced some transformant colonies. Donor DNA with 2,000-bp homology arms yielded 2.6 × 10^6^ CFU/mL of the transformants. Although this was the highest transformation efficiency in this study, excessively long homology arms can lead to issues such as incomplete amplification or PCR errors. Therefore, 500–1,000-bp homology arms are more manageable and practical for *P. gingivalis* mutagenesis.

### 3.5 Components of DNA uptake machinery in *Porphyromonas gingivalis*

Natural DNA competence involves a species-specific DNA uptake machinery that traverses the cellular envelope (Seitz and Blokesch, 2013). While most components of this machinery in *P. gingivalis* remain unknown, ComF (locus tag, PG0158 in W83; PGN_0270 in ATCC 33277), a cytoplasmic ATP-dependent DNA helicase, is the only component experimentally confirmed to be necessary for natural competence-mediated genetic transformation (Tribble et al., 2012). Based on *P. gingivalis* gene annotation by Kyoto Encyclopedia of Genes (KEGG; Kanehisa et al., 2023), we identified two additional proteins potentially involved in natural competence: PGN_0421 and PGN_0519. PGN_0421 contains a ComEA domain (COG accession no. COG1555) spanning positions 94–165 (*E*-value 6.03 × 10^−21^). ComEA is a DNA-binding protein with HhH motifs that facilitates DNA transport and is located in the periplasmic compartment in Gram-negative bacteria such as *Vibrio cholerae* (Matthey and Blokesch, 2016). On the other hand, PGN_0519 contains a ComEC domain (COG accession no. COG0658) spanning positions 213–488 (*E*-value 2.05 × 10^−41^). ComEC is a membrane protein that forms a channel in the cytoplasmic membrane to import extracellular DNA into the cytosol (Pimentel and Zhang, 2018).

**Figure 5:**
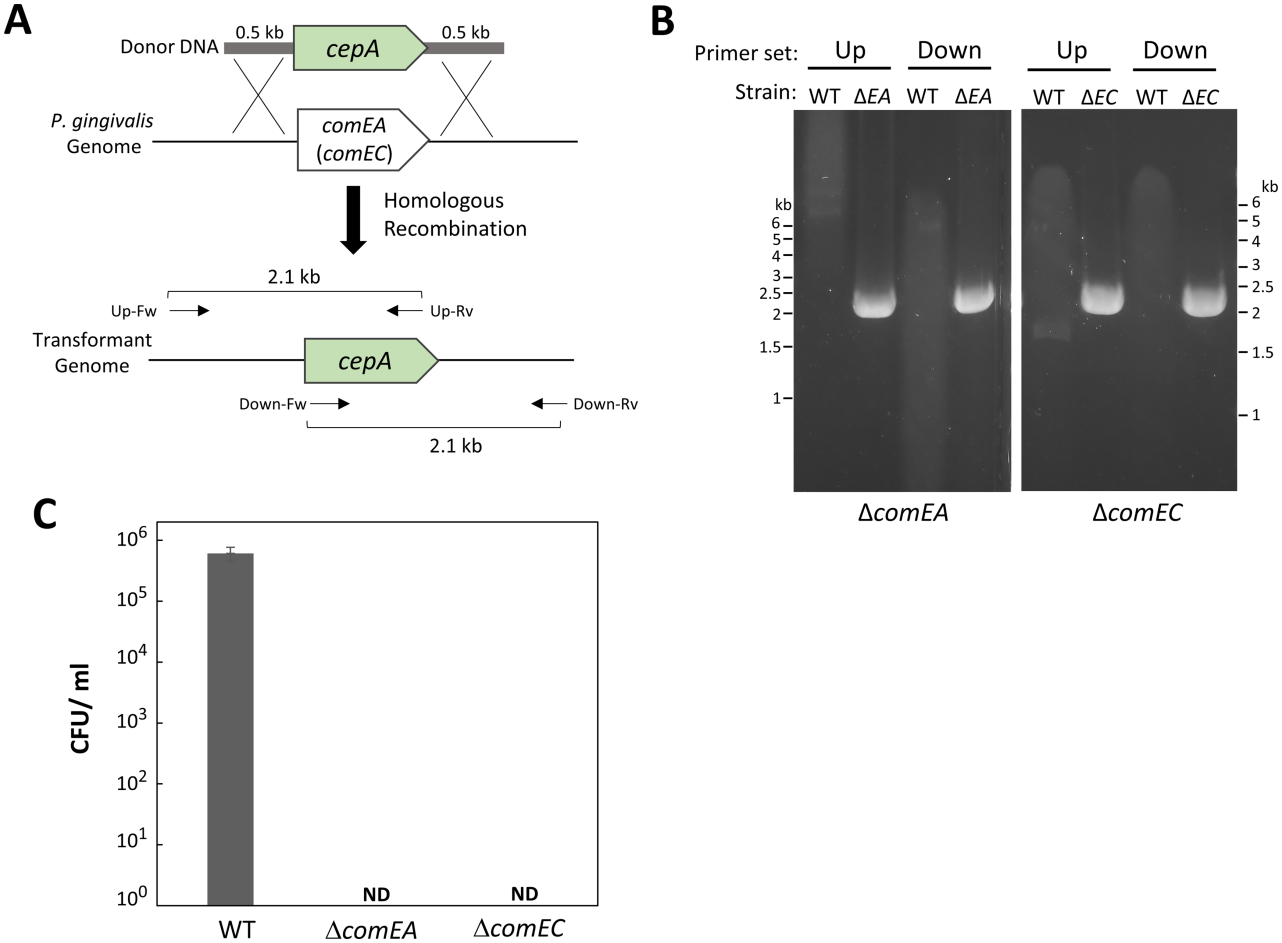
Gene deletions of *comEA* and *comEC* in *P. gingivalis* ATCC 33277. **(A)** Schematic of gene replacements for *comEA* (*PGN_0421*) and *comEC* (*PGN_0519*) with *cepA* via recombination. The *cepA* cassette contains 0.5-kb homology arms at the 5′ and 3′ ends corresponding to the upstream and downstream regions of *comEA* (or *comEC*). Arrows indicate the positions of PCR primers with the orientations used for genotyping. **(B)** PCR confirmation. The *comEA* and *comEC* deletion mutants were verified by PCR using combinations of *cepA*-specific and genome-specific primers: Up, upstream region-specific forward primer for *comEA* (or *comEC*) combined with *cepA*-specific reverse primer; Down, *cepA*-specific forward primer combined with downstream region-specific reverse primer for *comEA* (or *comEC*). WT, *P. gingivalis* ATCC 33277; ΔEA, Δ*comEA*; ΔEC, Δ*comEC*. **(C)** Transformation efficiency of *comEA* and *comEC* mutant strains. Transformation assays were conducted using 100 ng of *ermF* donor DNA with 1,000-bp homology arms under the same conditions as in Figure 1. Transformation efficiency was quantified as CFU/ml of Em^r^ colonies. Error bars represent ±standard deviations from three independent experiments. ND, Not Detected.

To investigate their involvement in natural competence, we constructed deletion mutants of *P. gingivalis comEA* (*PGN_0421*) and *comEC* (*PGN_0519*). As the donor DNA used in this study carried *ermF*, we replaced *comEA* and *comEC* with *cepA* (Figure 5A). Using our transformation method, we successfully generated the Δ*comEA* and Δ*comEC* mutants. Gene replacement with *cepA* was confirmed by PCR (Figure 5B). Transformation attempts with *ermF* donor DNA on the Δ*comEA* and Δ*comEC* mutants resulted in no colonies (Figure 5C), indicating that the transformation efficiency of these strains was below detectable levels. This finding suggests that ComEA and ComEC are essential for the natural competence of *P. gingivalis*.

### 3.6 Transformation of W83 and TDC60 strains

**Figure 6:**
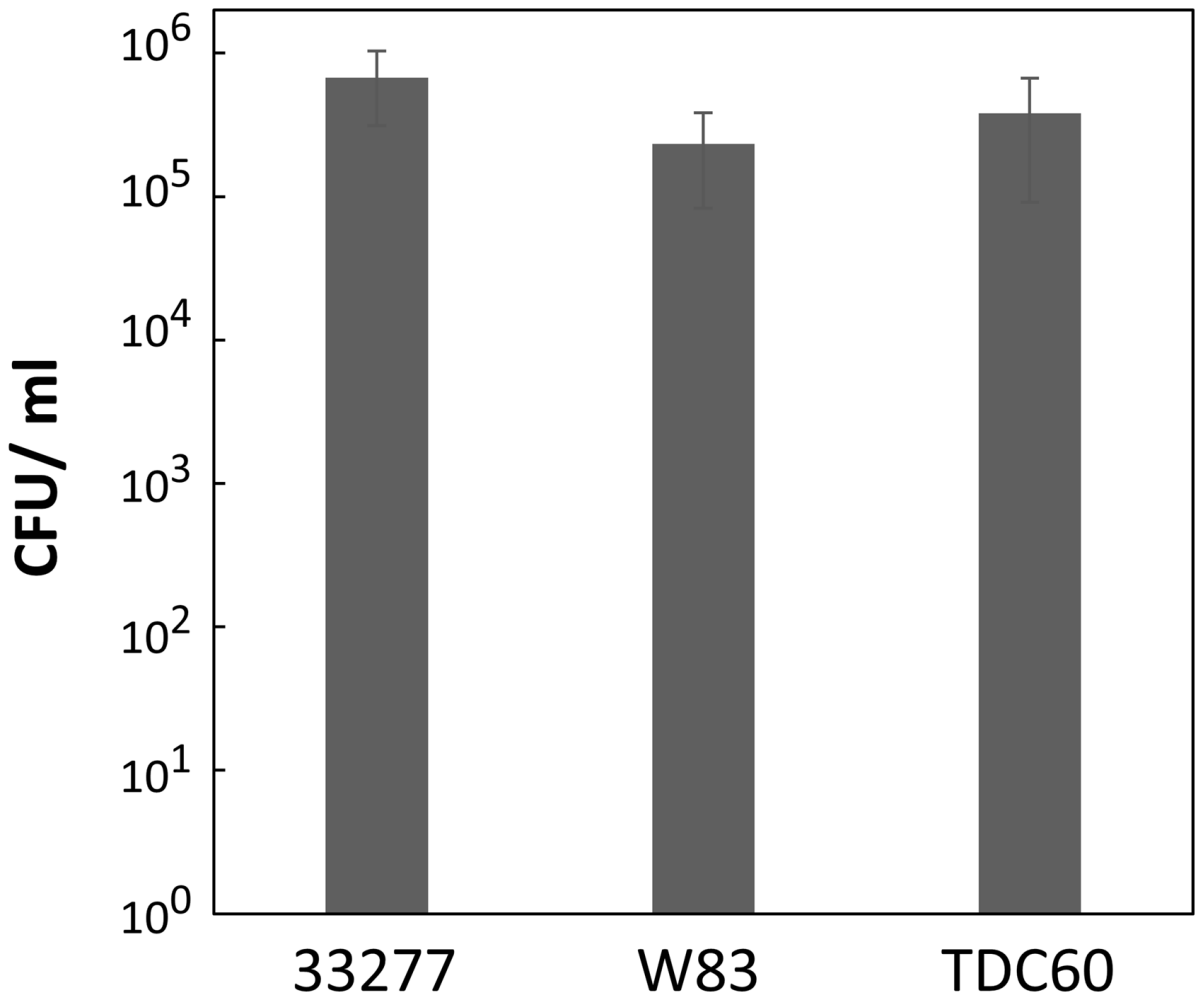
Transformation efficiency of *P. gingivalis* strains W83 and TDC60. Transformation assays of *P. gingivalis* ATCC 33277, W83, and TDC60 were conducted using 100 ng of *ermF* donor DNA with 1,000-bp homology arms under the conditions described in Figure 1. Transformation efficiency is shown as CFU/ml of Em^r^ colonies. Error bars represent ±standard deviations from three independent experiments.

This study exclusively used the ATCC 33277 strain; however, other type strains, such as W83 (Nelson et al., 2003) and TDC60 (Watanabe et al., 2011), are also commonly used in *P. gingivalis* research. To verify the DNA competency of these strains, we employed the strain-specific homology arms (1,000 bp each) for the *ermF* donor DNA to avoid nucleotide mismatches with ATCC 33277. *P. gingivalis* ATCC 33277, W83, and TDC60 were cultured to the exponential phase, mixed with 100 ng of donor DNA and allowed to develop biofilms for 24 h. The transformation assay revealed that W83 and TDC60 exhibited high DNA competency comparable to that of ATCC 33277, with no significant differences observed between the strains (Figure 6). In this assay, we used the same transformation conditions that were optimized for ATCC 33277. Since W83 and TDC60 have different characteristics, strain-specific adjustments could further enhance the transformation efficiency for W83 and TDC60. Overall, these results suggest that our transformation method is generally applicable in *P. gingivalis*.

## 4 Discussion

Natural competence is a physiological adaptation that allows bacteria to acquire new genetic traits by incorporation of exogenous DNA in response to environmental signals. Nutrient starvation and DNA damage—such as that caused by UV irradiation or mitomycin C treatment—are known to induce a competent state in many bacteria (Blokesch, 2016). We observed that *P. gingivalis* activates genetic competence during the early-exponential phase of liquid BHI-HM culture, even in the absence of DNA-damaging inducers (Figure 3B). This finding suggests that *P. gingivalis* responds to signals beyond starvation and DNA damage. Similarly, *Streptococcus pneumoniae* develops genetic competence during the early-mid growth phase through cell–cell communication using a peptide pheromone called CSP (competence stimulating peptide) (Salvadori et al., 2019). *P. gingivalis* may also utilize extracellular signaling molecules to regulate genetic competence. Additionally, high cell density, direct cell–cell contact, or biofilm matrix components could serve as competence-inducing signals, as colony biofilm enhances the genetic competence (Figure 3C).

Our natural competence-based method achieved the maximum efficiency of 7.7 × 10^6^ CFU/μg (Figure 3B, 10 h), allowing for transformation with as little as 10–100 ng of donor DNA (Figure 4A). In contrast, typical electroporation methods for *P. gingivalis* requires up to 1 μg of donor DNA per transformation (Bélanger et al., 2007). Given the high efficiency of natural competence, it is feasible to transform *P. gingivalis* by directly introducing a T4 ligase (or Gibson assembly) reaction mixture that includes upstream and downstream homology arms and an antibiotic resistance gene. In this study, we prepared the donor DNA using overlap extension PCR to assemble DNA fragments; however, this method often generated undesirable products. To enhance our methodology, future studies should consider the direct transformation of *P. gingivalis* with the ligation mixture. Moreover, we observed that transformation via natural competence produced transformants faster than electroporation, which can take up to 10 days (Bélanger et al., 2007). This difference is likely because natural competence does not damage the cells, whereas the high-voltage electric pulses generated by an electroporator can wound the cellular membrane and genomic DNA, impeding the cell growth and potentially inducing cell death. This characteristic of natural competence likely contributes to its high efficiency.

Another appealing aspect of natural competence for genetic engineering is the minimal restriction on the size of DNA that can be introduced. This is supported by the fact that chromosomal DNA is often used as donor DNA in many naturally competent bacteria, including *P. gingivalis* (Tribble et al., 2012). Such capability facilitates the genome-scale engineering of bacteria. For instance, Jagadeesh et al. successfully introduced *in vitro*-synthesized linearized DNA containing a 38-kb gene cluster for non-ribosomal peptide biosynthesis into *B. subtilis* competent cells (Jagadeesh et al., 2023). It is noteworthy that this was achieved using a strain lacking restriction and modification (RM) system, which exhibits two orders of magnitude higher competency than the parental strain (Uozumi et al., 1977). RM systems function as bacterial defenses against foreign DNA, such as bacteriophage genomes; however, they can present significant challenges for genetic engineering, particularly when introducing large DNA fragments. *P. gingivalis* ATCC 33277 also contains several putative RM enzymes (Naito et al., 2008). Inactivating these RM systems could be effective in introducing long donor DNA into *P. gingivalis*.

In addition to donor incorporation, the recombination process—integration of donor DNA into the host genome—is an important step in transformation. Recombineering, which utilizes bacteriophage-derived recombination factors, can be employed to enhance the recombination efficiency. For example, the lambda Red system facilitates efficient homologous recombination between donor DNA containing very short homology arms and the host genome (Brewster and Tolun, 2020). Site-specific phage integrases catalyze the accurate integration of donor DNA into a specific position in the host genome (Stark, 2017). These recombineering technologies would be beneficial for the genome-scale engineering of *P. gingivalis*.

Unlike conjugation and bacteriophage transduction, naturally competent cells actively transport foreign DNA into the cytosol. The DNA-uptake machinery is central to this process (Johnsborg et al., 2007). This study identified ComEA (PGN_0419) and ComEC (PGN_0512) as essential components for the natural competence (Figure 5). Based on their amino acids sequence homology, these proteins likely facilitate the translocation of DNA from the periplasmic space into the cytosol. On the other hand, the mechanism by which *P. gingivalis* imports exogenous DNA into the periplasmic space remains unclear. Typically, bacteria possess species-specific DNA-uptake machinery that resembles type II-or type IV-secretion systems (T2SS and T4SS) or type IV pilus (T4P) (Chen and Dubnau, 2004). In *P. gingivalis*, T2SS and T4SS have not been identified, and the T4P-like conjugation pilus of *P. gingivalis* is not involved in DNA uptake (Tribble et al., 2012). Recently, genetic screening systems based on next-generation sequencing technologies, such as transposon sequencing, have been developed (van Opijnen et al., 2009; Goodman et al., 2011). These cutting-edge technologies are highly effective and could significantly contribute to the genome-wide identification of factors involved in *P. gingivalis* natural competence.

The rise of antibiotic-resistant bacterial pathogens poses a significant threat to clinical and healthcare settings (Ajayi et al., 2024). Recent studies have highlighted the prevalence of antibiotic-resistant genes among oral pathogens, including *P. gingivalis* (Brooks et al., 2022). Horizontal gene transfer is a crucial mechanism driving the dissemination of antibiotic-resistant genes among bacteria, with natural competence being a major pathway (Abe et al., 2020). Understanding the natural competence of *P. gingivalis* will not only advance genetic engineering, but also help control the emergence of antibiotic-resistant oral pathogens.

## 5 Conclusion

In this study, we established a natural competence-based transformation method for *P. gingivalis*. Our approach is simple and efficient, allowing for the rapid generation of transformants. This technique enabled the insertion of antibiotic-resistant genes (Figures 2–4; Supplementary Figure S2) and gene deletion (Figure 5). Additionally, this method was successfully applied to strains W83 and TDC60 (Figure 6). Further research is needed to elucidate the mechanisms underlying the natural competence of *P. gingivalis*. This understanding will enhance genetic engineering of *P. gingivalis* and improve clinical applications.

## Data Availability

The original contributions presented in the study are included in the article/Supplementary material, further inquiries can be directed to the corresponding authors.
