# Supplementary material for: A simple and cost-effective transformation system for Porphyromonas gingivalis via natural competence
Source: Front Microbiol. 2024 Oct 21;15:1476171. doi: 10.3389/fmicb.2024.1476171 (PMC11532111; doi:10.3389/fmicb.2024.1476171)
Supplement: Supplementary file 1 [file Data_Sheet_1.PDF]

Supplementary Materials

A simple and Cost-effective Transformation System for *Porphyromonas gingivalis* via Natural Competence

Authors:
Kimihiro Abe, Hiroko Yahara, Ryoma Nakao, Takehiro Yamaguchi, Yukihiro Akeda.

Supplementary Tables

Table S1. Strains and plasmids.

Strain and plasmid	Genotype or relevant features	Source or reference
Strains		
Porphyromonas gingivalis		
ATCC 33277	Type strain, wild type strain in this study	Naito *et al*., 2008
Δ*comEA*	ATCC 33277 Δ*comEA*::*cepA*; Amp^r	This study
Δ*comEC*	ATCC 33277 Δ*comEA*::*cepA*; Amp^r	This study
KDP501	ATCC 33277 Δ*htrA*::*cepA*; Amp^r	Sato *et al*., 2018
W83	Type strain	Nelson *et al*., 2003
TDC60	Type strain	Watanabe *et al*., 2011
Plasmid		
pHS17	*E. coli-F. nucleatum shuttle verctor carrying ermF-ermAM*	Haake *et al*., 2000

Table S2. Primers used in this study.

Primer ID	Sequence (5′-3′)*	Purpose
PKB-301	ggcccaaacgcatggCTTCCGCTATTGCTTTTTTG	*ermF* amplification
PKB-302	cggccatctccttggTTTTACGTTTCCGCTCCATC	*ermF* amplification
PKB-417	AGTTTGTAGTTGGGATCGGGCAGC	amplification of 2,000-bp upstream arm of donor DNA
PKB-418	ccatgcgtttgggccTGAAAAACAGTCAATTGACAATG	amplification of upstream arms of donor DNA
PKB-419	ccaaggagatggccgGGTCTGTACAGATATAAATAC	amplification of downstream arms of donor DNA
PKB-420	GACGGTGTATCAGGAGCAAGTC	amplification of 2,000-bp downstream arm of donor DNA
PKB-421	ggcccaaacgcatggAAGCATCTTCGATGCTGGAG	*cepA* amplification
PKB-422	cggccatctccttggTAGTGATAGTGAACGGTTGT	*cepA* amplification
PKB-428	TTCGAGGATCATTTCGCCCC	amplification of 1,000-bp upstream arm of donor DNA for ATCC 33277 and W83
PKB-429	TCTCGCTCGAATAATCCGTA	amplification of 1,000-bp downstream arm of donor DNA for ATCC 33277, W83, and TDC60
PKB-440	ATACGACGAGTCCGGCAACT	amplification of 500-bp upstream arm of donor DNA
PKB-441	TATGGCACTGCAAATTACAG	amplification of 500-bp downstream arm of donor DNA
PKB-442	GACGTCATGTCAGCGAATCG	amplification of 250-bp upstream arm of donor DNA
PKB-443	GTAAAGAAGCTCGTGGGTGC	amplification of 250-bp downstream arm of donor DNA
PKB-444	GTTCGGGGAGTGGGAGGTTC	amplification of 100-bp upstream arm of donor DNA
PKB-445	CGGTTAGGCAACCGGCTTCA	amplification of 100-bp downstream arm of donor DNA
PKB-446	TGTCGCACGGCATTTTCCGG	amplification of 50-bp upstream arm of donor DNA
PKB-447	AAGACGTAGAAGCGAATCAG	amplification of 50-bp downstream arm of donor DNA
PKB-450	TCCGCCCGAAACATTGTTCA	PCR comfirmation for *comEC* (*PGN_0519*) deletion cassette
PKB-451	ccatgcgtttgggccTACCCACAGAAACGGTCCGG	amplification of upstream arm for *comEC* (*PGN_0519*) deletion cassette
PKB-452	ccaaggagatggccgAGTGACAAGCTGTCTCTCAT	amplification of downstream arm for *comEC* (*PGN_0519*) deletion cassette
PKB-453	TTTGCCGGACTCCGTTATCG	PCR comfirmation for *comEC* (*PGN_0519*) deletion cassette
PKB-454	AATCAGTTCGATGGGAGCAG	PCR comfirmation for *comEA* (*PGN_0421*) deletion cassette
PKB-455	ccatgcgtttgggccCTGTGTATCGGGGCTGTTTG	amplification of upstream arm for *comEA* (*PGN_0421*) deletion cassette
PKB-456	ccaaggagatggccgAATGATATGATCATCAAGAT	amplification of downstream arm for *comEA* (*PGN_0421*) deletion cassette
PKB-457	TCTACTCTTTCTTTAAGTCG	PCR comfirmation for *comEA* (*PGN_0421*) deletion
PKB-460	CGATGAAGTTCGTGGACAGG	amplification of upstream arm for *comEC* (*PGN_0519*) deletion
PKB-461	ACGCTCCGATTTTTATCGTT	amplification of downstream arm for *comEC* (*PGN_0519*) deletion
PKB-462	ACCGATATAATGGGCATGAA	amplification of upstream arm for *comEA* (*PGN_0421*) deletion
PKB-463	GTAAGACCATAGCGCTCTTC	amplification of downstream arm for *comEA* (*PGN_0421*) deletion
PKB-468	CCTGTACTTTGGGGTCGCCT	amplification of 1,000-bp upstream arm of donor DNA for TDC60
PKB-469	ccatgcgtttgggccACTATTTATATCTATATACA	amplification of 1,000-bp upstream arm of donor DNA for W83 and TDC60
PKB-470	ccaaggagatggccgGAGAGACTTGTATCAATCCT	amplification of 1,000-bp downstream arm of donor DNA for W83 and TDC60

*Engineered nucleotides are indicated by lower letters.

Supplementary Figures

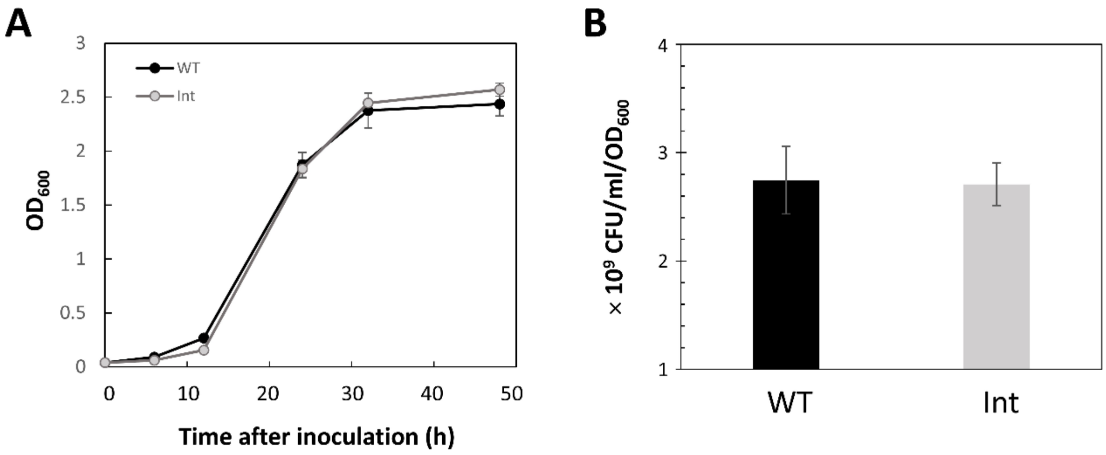

Figure S1. Cell growth and CFU of Em^r transformant. (**A**) Growth curve. Cell growth of the WT and Em^r transformant (Int) strains were monitored by OD_{600} measurement at the times indicated in the panel. The strains were cultured at 37 °C in liquid BHI-HM under anaerobic conditions. Black line, WT; gray line, Em^r transformant (Int). Error bars represent ±standard deviations from three independent experiments. (**B**) Cell viability. CFUs of the WT and Em^r transformant (Int) cultures at 48 h in the BHI-HM were normalized by their OD_{600} value. Error bars represent ±standard deviations from three independent experiments.

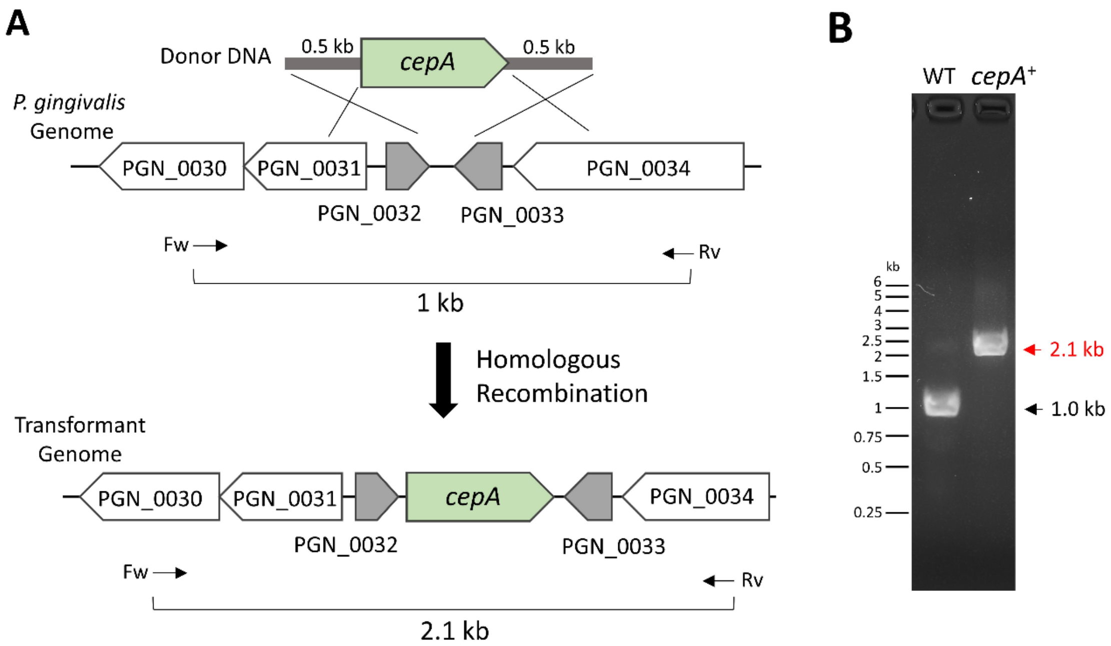

Figure S2. Integration of *cepA* donor DNA into *P. gingivalis* genome. (**A**) Schematic of integration of an ampicillin-resistant gene (*cepA*)-harboring donor DNA into the *P. gingivalis* ATCC 33277 genome through a double homologous recombination event. The *cepA* donor DNA was attached with 500-bp homology arms to be integrated into the intergenic region between *PGN_0032* and *PGN_0033*. Arrows denote the positions and directions of PCR primers for genotyping. (**B**) Colony PCR confirmation. The *cepA* integration was confirmed by colony PCR (*cepA*+), using the primers indicated in **A**. WT colony was used as a negative control.
